# Correlation between pain and degenerative bony changes on cone-beam computed tomography images of temporomandibular joints

**DOI:** 10.1186/s40902-017-0117-1

**Published:** 2017-07-05

**Authors:** SunMee Bae, Moon-Soo Park, Jin-Woo Han, Young-Jun Kim

**Affiliations:** 1 Department of Oral Medicine and Diagnosis, Research Institute of Oral Science, College of Dentistry, Gangneung-Wonju National University, 7 Jukhyun-gil, Gangneung, 25457 South Korea; 2 Department of Oral and Maxillofacial Radiology, Research Institute of Oral Science, College of Dentistry, Gangneung-Wonju National University, Gangneung, South Korea

**Keywords:** Temporomandibular joints (TMJ), Cone-beam computed tomography (CBCT), Degenerative bony change, Pain

## Abstract

**Background:**

The aim of this study was to assess correlation between pain and degenerative bony changes on cone-beam computed tomography (CBCT) images of temporomandibular joints (TMJs).

**Methods:**

Two hundred eighty-three temporomandibular joints with degenerative bony changes were evaluated. Pain intensity (numeric rating scale, NRS) and pain duration in patients with degenerative joint disease (DJD) were also analyzed. We classified condylar bony changes on CBCT into five types: osteophyte (Osp), erosion (Ero), flattening (Fla), subchondral sclerosis (Scl), and pseudocyst (Pse).

**Results:**

Degenerative bony changes were the most frequent in the age groups of 10~19, 20–29, and 50~59 years. The most frequent pain intensity was “none” (NRS 0, 34.6%) followed by “annoying” (NRS 3–5, 29.7%). The most frequent condylar bony change was Fla (219 joints, 77.4%) followed by Ero (169 joints, 59.7%). “Ero + Fla” was the most common combination of the bony changes (12.7%). The frequency of erosion was directly proportional to NRS, but the frequency of osteophyte was inversely proportional. The prevalence of Ero increased from onset until 2 years and gradually decreased thereafter. The prevalence of Osp, Ero, and Pse increased with age.

**Conclusions:**

Osp and Ero can be pain-related variables in degenerative joint disease (DJD) patients. “Six months to 2 years” may be a meaningful time point from the active, unstable phase to the stabilized late phase of DJD.

## Background

Temporomandibular disorder (TMD) refers to a collective term including clinical problems that involve the masticatory muscles, the temporomandibular joint (TMJ), and associated structures [1]. TMD is frequently associated with disc displacement and degenerative changes in the TMJ [2]. Degenerative joint disease (DJD) affects both soft and hard tissues including cartilage, subchondral bone, and synovial membrane. DJD can be diagnosed when there is either crepitus or radiographic bony changes [3]. Osteoarthrosis is also a DJD in which joint form and structures are abnormal but without signs of arthralgia [3]. DJD causes secondary synovial inflammation, TMJ remodeling, articular cartilage abrasion, and bone degradation characterized by development osteophytes, erosion, flattening, subchondral sclerosis, and pseudocysts [4, 5]. Detection and evaluation of these bony changes are fundamental for successful diagnosis of DJD [6]. The condition of TMJ can be evaluated by a variety of imaging modalities [7–9]. Cone-beam computed tomography (CBCT) is a fairly new imaging modality that can produce images of high diagnostic quality with a lower radiation dose than medical computed tomography [10–12].

It has been controversial whether degenerative bony changes of TMJ can be related to the onset, progression, or regression of TMJ-related signs and symptoms [2, 9]. The aim of this study was to assess the correlation between pain and condylar bony changes on CBCT images in DJD patients.

## Methods

### Subjects

Two hundred and fifty patients who visited the dental hospital from September 2013 to March 2015 complaining of TMJ pains, TMJ sounds, or mouth opening limitation were evaluated. Conventional radiological evaluations (panoramic view and transcranial view) and CBCT examinations were performed. Two hundred one patients (165 women and 36 men) with degenerative bony changes on their conventional radiographies and CBCT images were selected. We excluded normal condyles of DJD patients and finally evaluated 283 condyles.

Pain intensity and pain duration were examined. Pain intensity was evaluated with the numeric rating scale (NRS). We asked patients’ average pain intensity of the past 3 days. NRS is 0 to 10 verbal rating scale where “0” was no pain and “10” was the worst pain possible. We classified it into five grades (NRS 0, none; NRS 1–2, mild; NRS 3–5, annoying; NRS 6–7, bad; NRS 8–10, severe). In addition, we asked patients the onset time of their pain.

### Radiographic examination and evaluation

All subjects were scanned with Alphard-3030 (Asahi Roentgen Co., Kyoto, Japan) with P-mode, operating at 80 kV, 8 mA with a voxel of 0.30 mm. The primary reconstruction of the raw data was restricted to the TMJ region (approximately 3.5 cm superior to the mandibular fossa and 4 cm inferior, 4 cm anterior, and 3 cm posterior to the condylar neck) using Xelis dental program. The long axial view of the examined condyle was traced with the TMJ tool, and the software generated lateral and frontal cross-sectional reconstructions perpendicular and parallel to the long axis of the condyle, respectively. The thickness of the image slices was 1 mm, and the distance between slices was 1 mm for both lateral and frontal reconstructions. The reconstructed images were analyzed by three well-trained dentists. Right and left TMJs were examined separately, resulting in a total of 283 TMJs. Comparing the sagittal, coronal, and 3D images, we classified degenerative bony changes into five types: osteophytes, erosion, flattening, subchondral sclerosis, and pseudocysts [3]. For the accurate assessment, only the bony changes on the articular surfaces were evaluated. Condyles with hyperplasia, deviation in form, and systemic arthritis were excluded in this study. The criteria for the types of condylar bony change shows as follows:

- Osteophytes: marginal bony outgrowths on the condyle
- Erosion: an area of decreased density or discontinuity or irregularity of the cortical bone
- Flattening: a flat bony contour deviating from the convex form
- Sclerosis: an area of increased density of cortical bone extending into the bone marrow
- Pseudocysts: well-circumscribed osteolytic adjacent subcortical bone area without cortical destruction

### Statistical analysis

Simple regression analysis was used to assess the correlation of pain intensity and age on the occurrence of degenerative bone changes. *P* values less than 0.05 were considered statistically significant. Statistical evaluation of the data was performed using the IBM SPSS Statistics ver. 22.0 for Windows (IBM Co., Armonk, NY, USA)

## Results

This study was performed with 201 patients. The percentage of women (82%) were higher than men (18%) (Table 1). Subject age ranged from 12 to 81 years (mean 38 ± 19 years) (Table 2). Degenerative bony changes were the most frequent in the age groups of 10~19 years (25.4%), 20–29 years (19.9%) and 50~59 years (19.4%). We could not find an association between age and the prevalence of bony changes.

**Table 1:** Distribution of gender

	Patients	Degenerative condyles
	*n* (%)	*n* (%)
Women	165 (82)	237 (84)
Men	36 (18)	46 (16)
Total	201 (100)	283 (100)

**Table 2:** Distribution of age

	Patients	Degenerative condyles
	*n* (%)	*n* (%)
10~19	51 (25.4)	69 (24.4)
20~29	40 (19.9)	56 (19.8)
30~39	16 (8.0)	22 (7.8)
40~49	27 (13.4)	39 (13.8)
50~59	39 (19.4)	56 (19.8)
60~69	17 (8.5)	25 (8.8)
70~79	9 (4.5)	13 (4.6)
80~89	2 (1.0)	3 (1.1)
Total	201 (100)	283 (100)

**Fig. 1:**
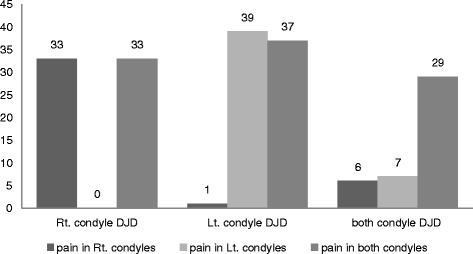
Prevalence of TMJ pain according to the bony changes. *DJD* degenerative joint disease, *Rt* right, *Lt* left, *TMJ* temporomandibular joint

Figure 1 shows the prevalence of TMJ pain according to the bony changes (left, right, or both). In the case of unilateral DJD, the TMJ pain on the degenerative condyle and the both condyles were similar (“33 vs. 33” and “39 vs. 37”). The prevalence of bony changes only in non-painful side was significantly low (0 and 1).

Table 3 presents distribution of degenerative condyles according to pain intensity. The most frequent pain intensity was “none” (NRS 0, 34.6%) followed by “annoying” (NRS 3–5, 29.7%). Table 4 presents distribution of degenerative condyles according to pain duration. About half of the degenerative condyles were examined at the hospital within 6 months after the pain had occurred.

**Table 3:** Distribution of degenerative condyles according to pain intensity

Pain intensity (NRS)	Degenerative condyles
	*n* (%)
None (0)	98 (34.6)
Mild (1, 2)	29 (10.2)
Annoying (3, 4, 5)	84 (29.7)
Bad (6, 7)	51 (18.0)
Severe (8, 9, 10)	21 (7.5)
Total condyle	283 (100)

*NRS* numeric rating scale

**Table 4:** Distribution of degenerative condyles according to pain duration

Pain duration	Degenerative condyles
	*n* (%)
~6 months	90 (48.6)
6 months~1 year	16 (8.6)
1~3 years	28 (15.1)
3~10 years	40 (21.6)
Over 10 years	11 (5.9)
	185 (100)

**Fig. 2:**
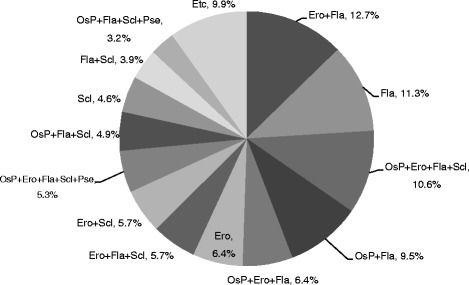
Distribution of degenerative bony change in degenerative condyles. *Osp* osteophyte, *Ero* erosion, *Fla* flattening, *Scl* sclerosis, *Pse* pseudocyst

Accumulative number and percentage of degenerative bony change in degenerative condyles are presented in Table 5. When the multiple radiographic findings were detected in one condyle, each finding was counted separately. The most frequent bony change was “flattening” (219 joints, 77.4%) followed by “erosion” (169 joints, 59.7%), and “sclerosis” (139 joints, 49.1%). Figure 2 shows the distribution of degenerative bony change in degenerative condyles. The combination of erosion and flattening (“erosion + flattening”) was the most common bony change (12.7%). The proportions of degenerative changes detected as combined forms were 77.0%.

**Table 5:** Accumulative number and percentage of degenerative bony change in degenerative condyles

Bony change	Degenerative condyles
	*n* (%)
Osteophytes	128 (45.2)
Erosion	169 (59.7)
Flattening	219 (77.4)
Sclerosis	139 (49.1)
Pseudocysts	43 (15.2)
Total	698

**Fig. 3:**
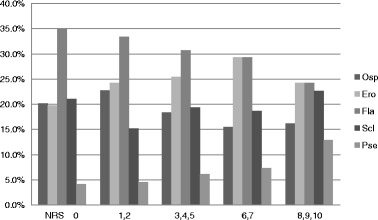
Distribution of types of bone change according to pain intensity. *NRS* numeric rating scale, *Osp* osteophyte, *Ero* erosion, *Fla* flattening, *Scl* sclerosis, *Pse* pseudocyst

**Fig. 4:**
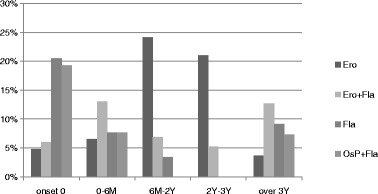
Distribution of types of bone change according to pain duration. *Onset 0* asymptomatic condyles, *M* month, *Y* year, *Osp* osteophyte, *Ero* erosion, *Fla* flattening

Figure 3 presents distribution of types of bone change according to pain intensity. According to the simple regression analysis, pain intensity (categorized into NRS group) showed a statistically significant correlation with “osteophyte” and “erosion” (Table 6). The frequency of erosion was directly proportional to pain intensity, but the frequency of osteophyte was inversely proportional (Fig. 3). Because of insufficient sample size, the NRS 8–10 group was excluded from data analyses. Flattening was the most frequent type (35%) in none group (NRS 0). Erosion and flattening were the most prevalent changes in all groups except NRS 0 group. The prevalence of erosion increased from onset until 2 years and gradually decreased thereafter (Fig. 4).

**Table 6:** Result of the simple regression analysis for the presence of degenerative bony changes (osteophyte, erosion) based on pain intensity (categorized into NRS group)

	Coefficient	Standard error	*t* score	*p* value	95% confidence interval (range)
Erosion	1.405	0.332	4.236	0.051	−0.02 to 2.83
Osteophyte	−0.739	0.219	−3.377	0.043	−1.436 to −0.043

**Fig. 5:**
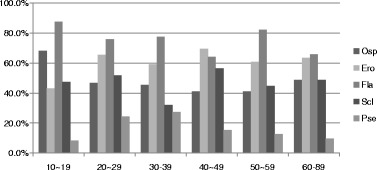
Distribution of types of bone change according to age. *Osp* osteophyte, *Ero* erosion, *Fla* flattening, *Scl* sclerosis, *Pse* pseudocys

Figure 5 shows the distribution of types of bone change according to age. According to the simple regression analysis, age showed a statistically significant correlations with the presence of several bone changes (“osteophyte,” “erosion,” and “pseudocyst”) (Table 7). The prevalence of osteophyte, erosion, and pseudocyst increased with age. Flattening was the most common radiographic finding in all age group. Pseudocyst was observed more in 60–81 years old (12.6%) than in other age groups.

**Table 7:** Result of the simple regression analysis for the presence of degenerative bony changes (erosion, osteophyte, pseudocyst) based on age groups

	Coefficient	Standard error	*t* score	*p* value	95% confidence interval (range)
Erosion	0.517	0.095	5.425	0.006	0.252 to 0.782
Osteophyte	0.634	0.155	4.092	0.015	0.204 to 1.065
Pseudocyst	0.473	0.122	3.864	0.018	0.133 to 0.813

## Discussion

Several studies reported that the progression and the severity of bony changes on the TMJ increased with age [13–15]. On the other hand, our study revealed that the prevalence of bony changes was higher in the groups of 10~19, 20–29, and 50~59 years old than in other age groups (Table 2). This finding was consistent with the former study [16] that bony changes were more frequent between 20 and 49 years old. Because psychologic status can be a precipitating factor of TMD [17, 18], our finding may be partially related to the stress and the pressure of study for the college entrance examination in Korean teenagers. In addition, young people have a tendency to visit a hospital more often than aged people (Table 2).

The intermediate phase of bony destruction in TMJ lasts on average 6 months to 1 year [15]. In the present study, 48.6% of degenerative condyles were examined within 6 months. We speculated that patients usually visited the hospital in their intermediate phase of DJD, when they might undergo spontaneous joint pain, mouth opening limitation, and/or crepitus [5].

Generally, bone deformation characterized in DJD is osteophytes, erosion, flattening, sclerosis, and pseudocysts. Each type of bony change occurs in different stages of DJD and has different clinicopathological meanings [11]. As a condyle has adapted to degenerative changes, tissue remodeling has happened and radiographic and/or morphologic appearances of condyles have changed accordingly [12]. Several papers reported the distribution of condylar bony changes and their combinations [10, 13, 14]. These studies presented somewhat different results with the others. Dos Anjos Pontual et al. found flattening to be the predominant findings [13]. Wiese et al. found “flattening + osteophyte + erosion” to be the predominant findings [14]. Campos et al. reported that “osteophytes + erosion” was the most frequent combination and osteophyte was the most common single bony change in the MRI study [10]. We found that erosion + flattening was the most frequent (12.5%), followed by “flattening” (11.5%), “osteophyte + erosion + flattening + sclerosis” (10.4%), and “osteophyte + flattening” (10.1%). The reason for these different results among the studies may be that it was not easy to detect the bony changes definitively, since it is usually a gradual remodeling process [10, 13, 14]. As CBCT has been widely used in assessing TMJ morphology, more specific or detailed guidelines for degenerative bony changes are necessary [19].

Even though pain has occurred only in one side, degenerative condylar changes can be detected on both sides (Fig. 1). One third of degenerative condyles did not show pain (Table 3). These results can imply that degenerative changes show some degree of inflammation, producing symptoms that resolve with time, while the previous bony changes still remain [10].

Erosion is a radiographic clue that an active destructive process may be occurring, whereas osteophyte is an indication that the condyle has adapted to degenerative changes produced in the past [11]. In this study, the frequency of erosion was directly proportional to NRS, but the frequency of osteophyte was inversely proportional (Fig. 3). This result demonstrates that the active inflammation of DJD is correlated with the erosion and inversely correlated with the osteophyte.

The prevalence of erosion increased from onset until 2 years and gradually decreased thereafter (Fig. 4). This result suggests “6 months to 2 years” might be a meaningful time point when DJD status changes from the active, unstable phase to the stabilized late phase. The prevalence of osteophyte, erosion, and pseudocyst was increased with age (Fig. 5). Considering these results, we suppose that erosion would have remodeled into osteophyte and/or pseudocyst, as time goes by.

Whereas some previous studies reported that there was poor correlation between bony change and pain [12, 14], our study found that osteophyte and erosion could be pain-related variables in DJD. We speculate that these contradictory results may be due to considering only the existence of bony changes, not the type of bony change in the previous study. There were still controversies about correlation between pain and condylar bony changes [9, 12, 14, 20].

Our study has several limitations such as the limited sample size. Moreover, we evaluated only the first-visit results and excluded the follow-up results. To verify the significant relationships between pain and radiographic findings, further well-organized studies will be needed in the future.

## Conclusions

The present study may find a correlation between pain intensity and degenerative bony changes on CBCT images. Osteophyte and erosion can be pain-related variables in DJD patients. Six months to 2 years may be a meaningful time point from active, unstable phase to stabilized late phase of DJD.

## Abbreviations

CBCT: Cone-beam computed tomography
DJD: Degenerative joint disease
Ero: Erosion
Fla: Flattening
NRS: Numeric rating scale
Osp: Osteophyte
Pse: Pseudocyst
Scl: Subchondral sclerosis
TMD: Temporomandibular disorder
TMJ: Temporomandibular joints
